# Tobacco production under global climate change: combined effects of heat and drought stress and coping strategies

**DOI:** 10.3389/fpls.2024.1489993

**Published:** 2024-11-26

**Authors:** Ming Liu, Xianglu Liu, Yuxiao Song, Yanxia Hu, Chengwei Yang, Juan Li, Shuangzhen Jin, Kaiyuan Gu, Zexian Yang, Wenwu Huang, Jiaen Su, Longchang Wang

**Affiliations:** ^1^ College of Agronomy and Biotechnology, Southwest University/Engineering Research Center of South Upland Agriculture, Ministry of Education, Chongqing, China; ^2^ Institute of Grain Crops, Agricultural Science Extension Research Institute of Dali Bai Autonomous Prefecture, Dali, Yunnan, China; ^3^ Dali Prefecture Branch of Yunnan Tobacco Company, Dali, Yunnan, China; ^4^ Yunnan Agricultural University, Kunming, Yunnan, China

**Keywords:** high temperature, limited water irrigation, interactive role of high temperature and water stress, plant growth-yield-quality, physiological mechanisms, tobacco

## Abstract

With the intensification of global climate change, high-temperature and drought stress have emerged as critical environmental stressors affecting tobacco plants’ growth, development, and yield. This study provides a comprehensive review of tobacco’s physiological and biochemical responses to optimal temperature conditions and limited irrigation across various growth stages. It assesses the effects of these conditions on yield and quality, along with the synergistic interactions and molecular mechanisms associated with these stressors. High-temperature and drought stress induces alterations in both enzymatic and non-enzymatic antioxidant activities, lead to the accumulation of reactive oxygen species (ROS), and promote lipid peroxidation, all of which adversely impact physiological processes such as photosynthetic gas exchange, respiration, and nitrogen metabolism, ultimately resulting in reduced biomass, productivity, and quality. The interaction of these stressors activates novel plant defense mechanisms, contributing to exacerbated synergistic damage. Optimal temperature conditions enhance the activation of heat shock proteins (HSPs) and antioxidant-related genes at the molecular level. At the same time, water stress triggers the expression of genes regulated by both abscisic acid-dependent and independent signaling pathways. This review also discusses contemporary agricultural management strategies, applications of genetic engineering, and biotechnological and molecular breeding methods designed to mitigate adverse agroclimatic responses, focusing on enhancing tobacco production under heat and drought stress conditions.

## Introduction

With the intensification of global climate change, the frequency and intensity of extreme weather events are gradually increasing, with heat and drought stress becoming the primary abiotic stress factors affecting plant growth, development, productivity and crop quality (Hossain et al., 2012; Lobell et al., 2015). According to the Sixth Assessment Report of the Intergovernmental Panel on Climate Change (IPCC), global temperatures have increased by approximately 0.69–1.08°C over the past century, with projections indicating a further rise of 0.3–4.8°C by the end of the 21st century. Concurrently, issues related to water scarcity and their uneven distributions have exacerbated the prevalence of drought. Tobacco is a significant economic cash crop, cultivated worldwide, and has various products, including cigarettes, cigars, and pipe tobacco (Van Liemt, 2001). The tobacco industry plays a vital role in enhancing national revenue and gross domestic product (GDP), fostering local economic development, and increasing tobacco farmers’ income while exerting a substantial influence on the global economy. However, tobacco production is susceptible to adverse agroclimatic conditions, and climate change has significantly affected its production with significant challenges (Fahad et al., 2017).

**Figure 1:**
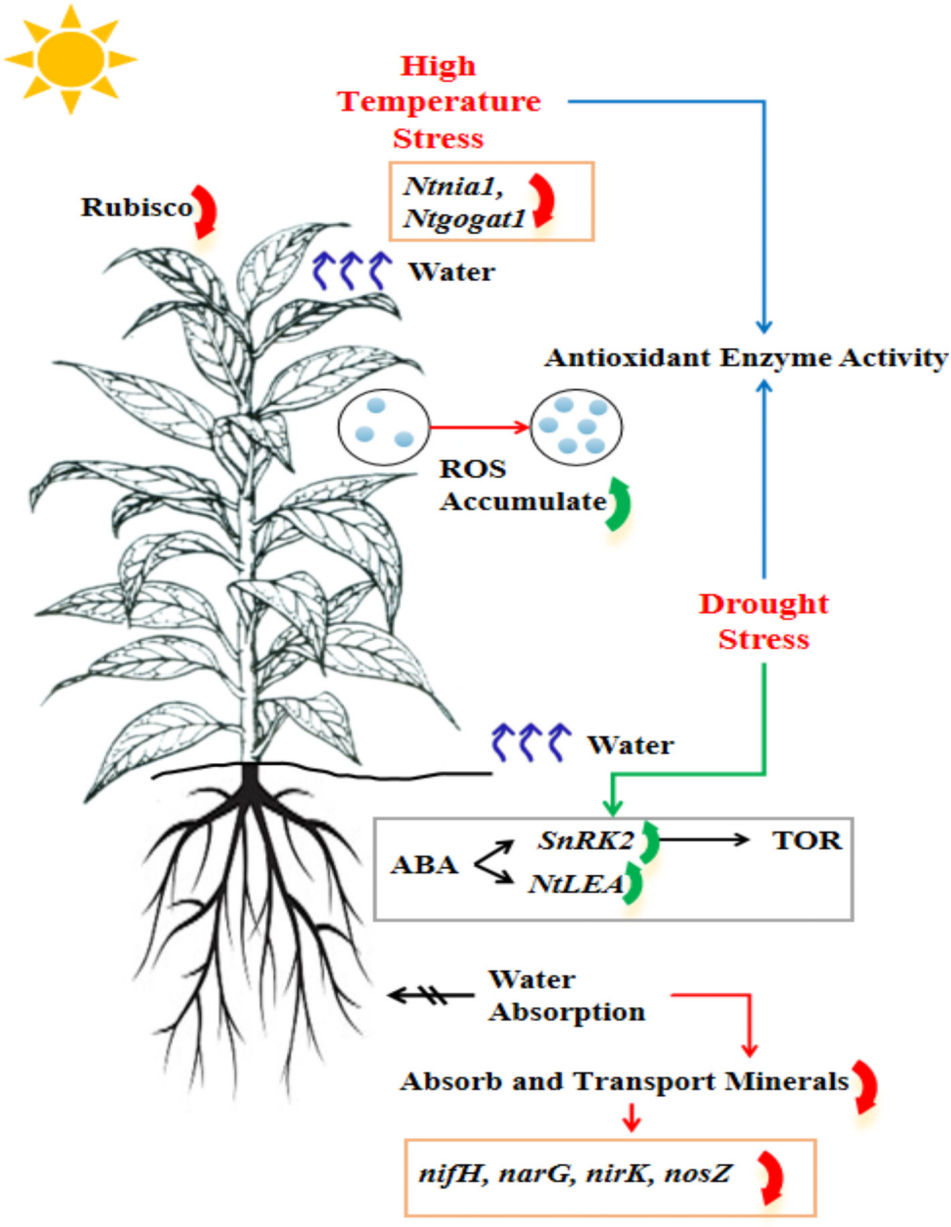
Influence of high temperature and limited water supply on tobacco plant production to tolerate these stress conditions.

Heat and drought are the most common abiotic stress, significantly affecting physiological and metabolic processes and downregulating photosynthetic efficiency (Mohammadi et al., 2024). These stresses induce oxidative stress, leading to lipid peroxidation of cell membranes, protein denaturation, and nucleic acid damage, triggering molecular and biochemical responses (**Figure 1**). In recent years, extensive research has been conducted to investigate the effects of heat and drought stress on tobacco production and the underlying physiological mechanisms. This research encompasses various dimensions, including growth, development, physiological responses, yield, and quality (Farooq et al., 2009; Zhou et al., 2017; Hu et al., 2023). However, heat and drought stress often co-occur in practical agricultural production, exhibiting more complex interactions and synergistic effects, exacerbating plant stress responses and leading to more severe physiological damage and yield loss. The synergistic effects of heat and drought stress have not yet been fully explored (Lamaoui et al., 2018). Consequently, this article examines current research findings regarding the physio-biochemical and molecular mechanisms of action in response to heat and drought stress at various growth stages of tobacco plants and their effects on yield and quality. However, it discusses strategies for mitigating stress to enhance tobacco production’s resilience to heat and drought in the context of global climate change.

## Effects of heat and drought stress on the growth and development of tobacco plants

Severe drought stress and prolonged exposure to temperatures exceeding the optimal range can damage plant growth and development (Lipiec et al., 2013). Tobacco plants exhibit differential responses to temperature variations at different developmental stages. Specifically, when temperatures surpass 30°C, tobacco plants are susceptible to leaf scorching during the seedling stage, which manifests as dark yellow and wrinkled foliage (Pearce et al., 2015). During the rosette to vigorous growth stages, continuous high temperatures cause wilting, drooping, and scorching of the upper plant leaves, with the leaf tissue on one side of the midrib showing wilting, deformity, or necrotic spots. In severe conditions, the entire plant can wilt and die (Bittner et al., 2016). In the late growth stage, high temperatures reduce the plant growth rate, reduce the stratification of leaf yellowing, and may even cause premature ripening (Li et al., 2021).

Tobacco plants require sufficient water throughout their entire growth period, with different stages demanding varying water levels (Driedonks et al., 2016). The soil moisture below 50% of the field capacity affects tobacco growth and development, reducing yield and quality (Prasad et al., 2008). Insufficient soil moisture reduces the water absorption capacity of tobacco seeds, thereby downregulating metabolic and enzymatic activities, which extends germination time and frequency (Chen et al., 2016). Research findings showed that water deficiency significantly reduces the seed germination frequency and vigor index (Abrantes et al., 2019). However, agronomic traits such as seedling and root length and fresh and dry biomass are significantly reduced in response to drought stress than control conditions (Su et al., 2017). Research results indicate that if tobacco seedlings experience water stress during transplanting, their photosynthetic CO_2_ assimilation rate will significantly downregulate (Synková and Valcke, 2001). During the rooting stage, mild drought conditions lead to rapid root volume growth, enhanced fresh and dry mass, root activity and dry matter accumulation (Aharon et al., 2003). However, drought significantly inhibits root development during vigorous growth, resulting in a noticeable decline in root-related indicators and slowdown of dry matter accumulation (Kumar et al., 2021). Severe water shortage at any growth stage leads to poor root development, with dry matter being allocated higher to the roots than aboveground parts, increasing the root-to-shoot ratio (Martiniello and Teixeira da Silva, 2011). Tang et al. (2021) demonstrated that soil moisture content (SMC) positively correlates with the number of tobacco leaves and leaf area expansion. Insufficient moisture negatively impacts leaf development by reducing the total number of leaves, inducing premature ageing of lower leaves, causing upper leaves to shrink and thicken, and roughening leaf tissue. Additionally, it diminishes stem circumference, impedes the growth of stem dry weight, and significantly reduces overall dry matter accumulation.

## Role of physiological and biochemical responses during growth and development stage

Photosynthesis is widely recognized as one of the most sensitive physiological processes in response to heat stress (Hasanuzzaman et al., 2013a). Under stress conditions, the main limiting factors for photosynthesis are disruptions in chloroplast function and reduced efficiency of the photosynthetic system (Salvucci and Crafts-Brandner, 2004; Wang et al., 2012). The optimum temperature significantly inhibits photosynthesis in tobacco leaves (Yanhui et al., 2020). Even under the same light conditions, the transpiration rate, stomatal conductance, and photosynthetic intensity of the upper leaves of the Yunyan 87 variety under heat treatment were significantly downregulated. At the same time, the activities of critical enzymes in the xanthophyll cycle, such as violaxanthin de-epoxidase and zeaxanthin epoxidase, were also reduced (Martín et al., 2015). It suggests that the impact of high temperatures on photosynthesis is primarily reflected in the inhibition of enzyme activities related to photosynthesis in the leaves (Song et al., 2014). Under moderate stress, photosynthetic carbon assimilation is the first to be affected, mainly due to the reduced activity of ribulose-1,5-bisphosphate carboxylase/oxygenase (Rubisco). At the same time, the impact on photosystem II (PSII) is relatively minor (Mathur et al., 2014). Heat stress can cause irreversible damage to the photosystem, disrupt the electron transport chain (ETC) and thylakoid membrane structure, and decline photosynthetic efficiency (Ergo et al., 2018). When temperatures reach a certain threshold, PSII suffers irreversible damage, the thylakoid membrane structure is disrupted, and the electron transport process becomes disordered (Yan et al., 2012). Prolonged exposure to high temperatures can lead to the death of cells, leaves, and entire plants (Feller and Vaseva, 2014).

Additionally, heat stress directly affects the activity of respiratory enzymes, reducing respiration intensity (Mathur et al., 2014). The intercellular CO_2_ concentration in tobacco cultivar Yunyan 87 initially decreases, then increases, and decreases again, indicating that prolonged heat stress weakens tobacco leaf respiration (Hu et al., 2021). During drought condition, stomatal limitations and reduced CO_2_ assimilation enzyme activities are the main factors causing to decline photosynthetic responses (Fahad et al., 2017; Zandalinas et al., 2018). Tobacco plant leaves typically exhibit noticeable yellowing and chlorosis, and as the severity increases, chlorophyll gradually degrades, leading to red or brown spots on the leaf surface (Raina et al., 2021). Drought stress significantly reduces chlorophyll content in tobacco leaves, thereby adversely impacting photosynthetic efficiency (Qi et al., 2023).The reduction in photosynthetic intensity under drought stress is attributed to stomatal and non-stomatal limitations. During mild drought conditions, the reduction in photosynthetic rate is primarily due to stomatal limitations. Insufficient water causes stomatal closure, hindering CO_2_ uptake and reducing photosynthesis due to inadequate raw materials. When tobacco plants are exposed to moderate or severe drought for extended periods, the decline in photosynthetic rate is due to the combined effects of both factors (Yan et al., 2024).

Under adverse climatic conditions, antioxidant enzyme activities are affected, leading to excessive accumulation of reactive oxygen species (ROS) in cells, which induces lipid peroxidation of membranes, disrupts membrane lipids and membrane proteins, increases membrane permeability, and damages membrane structural integrity in tobacco plants. This leads to protein and nucleic acid denaturation, causing plant metabolic disorders (Che et al., 2024). Under heat stress, the H_2_O_2_ content in tobacco leaves upregulated significantly, along with an increase in malondialdehyde (MDA) content. However, the activities of superoxide dismutase (SOD) and peroxidase (POD) activities are significantly enhanced. Excessive POD activity may enhance the POD-H_2_O_2_ decomposition system, promoting chlorophyll degradation, which decreases chlorophyll content (Tan et al., 2020). Different drought frequencies have varying effects on the activities of SOD, POD and catalase (CAT) in tobacco plant leaves. Under drought stress, SOD activity initially increases and then gradually decreases according to time and duration of stress frequency (Zhang and Kirkham, 1994; Li-Ping et al., 2006)). The type of tobacco varieties and degree and duration of stress affect the extent of cell membrane damage (Rizhsky et al., 2002; Li et al., 2011). Therefore, when studying stress resistance mechanisms, it is essential to consider and analyze multiple factors comprehensively.

## Impact of heat and drought stress on biomass, yield and quality

Heat and drought stress significantly affect tobacco biomass and yield (Dobra et al., 2010). High-temperature conditions severely inhibit the growth and development of tobacco plants, leading to diminished biomass and production (Yang et al., 2018). These stressors adversely affect physiological processes, including photosynthesis, respiration, and water metabolism, ultimately weakening growth potential and reducing dry matter accumulation (Wahid et al., 2007). Optimal temperature ranges prompt stomata closure in tobacco leaves, diminishing the plant’s capacity to absorb CO_2_ and inhibiting photosynthetic responses. This reduction in photosynthetic efficiency directly affects the accumulation of photosynthetic products, further decreasing tobacco biomass and disrupting water metabolism (Demirevska et al., 2010). Under elevated temperatures, the transpiration rate and water loss are accelerated, and the roots’ ability to absorb water diminishes, resulting in an insufficient water supply within various plant structures (Ben-Yehoshua and Rodov, 2002). Water deficit adversely affects cell turgor, constraining cell division and expansion, which ultimately leads to a reduction in tobacco biomass. This decline in biomass results in limited leaf area expansion and fewer leaves, affecting the number of harvestable tobacco leaves (Razi and Muneer, 2021; Fang and Xiong, 2015).

Furthermore, elevated temperatures are often encountered during the maturity stage of tobacco cultivation. Under such conditions, tobacco leaves may experience “forced ripening” due to thermal stress, resulting in the premature yellowing of three or more leaves on a single plant. In severe cases, the entire plant may exhibit abnormal yellowing. Under optimal soil moisture conditions, the yield per plant and acre of tobacco is significantly higher than drought-stress conditions (Moore and Tyson, 1999). Drought stress affects the development of tobacco plant roots. Water is a crucial factor for root growth, and under water-deficient conditions, root growth is hindered, leading to reduced water and nutrient absorption capacity (Bodner et al., 2015). The weakening of root function affects water regulation within the plant and the absorption and transport of minerals, thereby limiting plant growth and biomass accumulation. Dry soil and air conditions result in nutrient deficiency and slow development in plants, with the most obvious manifestation being a slowdown in leaf growth (Lawlor et al., 1981). The impact of drought stress on different parts of the tobacco leaves also varies, with the middle leaves being more affected than the upper leaves (Cakir and Cebi, 2010). Drought-induced cell dehydration inhibits leaf cell growth and leaf area expansion (Alves and Setter, 2004). Current research found that the quality of upper and lower tobacco leaves is significantly reduced during drought stress compared to control conditions (Dobra et al., 2010). The impact of drought stress on tobacco yield also varies with the growth stage. Moderate stress during the rooting is beneficial for root development, while the vigorous growth stage is a critical period for water, and water deficiency during this stage significantly reduces yield and quality. Slightly lower water levels during maturity can improve leaf quality, but severe water deficiency or excess water can reduce quality.

## Effects on tobacco leaf quality

Heat and/or drought or combined stress severely affects the quality of tobacco leaves (Hasanuzzaman et al., 2013b; Su et al., 2017). High temperatures can disrupt the balance of carbon and nitrogen metabolism in tobacco plants (Glaubitz et al., 2015). Due to the inhibition of photosynthesis, the synthesis of carbohydrates reduces while respiration increases, consuming more carbohydrates and leading to the reduction in starch content in tobacco leaves. High temperatures stimulate an increase in nicotine content in tobacco leaves. However, the potassium content in tobacco leaves positively correlated with field temperature (Chen et al., 2016). High-temperature treatment significantly accelerates the degradation of soluble proteins in tobacco leaves and increases the MDA content, thereby accelerating tobacco plants’ ageing. Different tobacco varieties respond differently to heat stress, resulting in variations in the chemical composition of tobacco leaves (Leffingwell, 1999; Yang et al., 2018). For example, the yield and quality of the K326 and Yunyan 87 tobacco varieties are mainly influenced by water factors. In contrast, temperature factors primarily affect the yield and quality of the Hongda variety of tobacco.

Under drought stress, tobacco quality typically declines, manifested by the reduction in sugar content and increase in nicotine content. Researchers found that under non-irrigated conditions, the average sugar content dropped about 5% over three years, resulting in poor leaf quality and reduced economic value. The average nicotine content over three years ranged from 1.7 to 2.72%, with no significant impact on nicotine content except under non-irrigated conditions (Biglouei et al., 2010). Water scarcity limits photosynthesis and carbohydrate synthesis. At the same time, drought stress alters metabolic pathways in plants, diverting more nitrogen into alkaloid synthesis (Singh et al., 2018). It also affects the content of minerals like potassium, calcium, and magnesium in tobacco leaves. Typically, these elements’ absorption and transport capacity decreases under drought conditions, reducing mineral content in tobacco leaves. It directly affects the combustibility and flavor of the tobacco leaves. Even when plants grow in nutrient-rich soils, drought can cause nutrient deficiencies by directly affecting the physical and chemical properties of the soil, reducing nutrient mobility in the soil and plant nutrient uptake (Ahanger et al., 2016). During tobacco growth and development, drought at different stages can affect tobacco leaf quality to varying degrees, including chemical composition and aromatic substances. For example, drought during vigorous growth significantly impacts tobacco leaf yield and quality, downregulating biomass, reducing sugar content and increasing total nitrogen and nicotine content (Su et al., 2017).

## Synergistic effects of heat and drought stress

The combined effects of heat and drought stress on plants cannot be attributed to the sum of the individual effects of stress; instead, they trigger new defense mechanisms in plants (Rizhsky et al., 2004). Under the combined stress, the physiological responses of tobacco plants exhibit high complexity (Wang et al., 2024). Water deficit is significantly exacerbated when plants experience combined heat and drought stress. High temperatures increase transpiration rates, while drought reduces soil water availability, preventing plants from obtaining sufficient water through their roots (Bodner et al., 2015; da Silva et al., 2010). A significant imbalance between water supply and demand disrupts water metabolism within the plant, resulting in decreased cell turgor, leaf wilting, and potentially irreversible damage. This condition ultimately constrains crop growth and productivity (Wollenweber et al., 2010; Gourdji et al., 2013).

Optimum temperature causes the stomatal functioning of tobacco plants to close, reducing water evaporation and limiting CO_2_ absorption (Driesen et al., 2020). Under drought conditions, the reduction in soil moisture further exacerbates stomatal closure, decreasing CO_2_ availability, inhibiting photosynthetic electron transport, lowering leaf photochemical efficiency of PSII, and ultimately reducing photosynthesis. This limitation on photosynthetic productivity downregulates the accumulation of assimilates (Martin-StPaul et al., 2017). Additionally, combined heat and drought stress enhances transpiration rates, accelerating water loss within the plant, which affects the utilization of limited soil moisture and exacerbates the challenges in crop growth and yield formation (Xu et al., 2013).

Key enzymes in nitrogen metabolism, such as nitrate reductase (NR) and glutamine synthetase (GS), played a critical role in assimilating ammonium into organic compounds (Xiong et al., 2018; Tao et al., 2018). Heat and drought stress severely inhibit nitrogen metabolism in plants, reducing NR activity, impairing nitrogen metabolism and absorption, and leading to reduction in nitrogen content in tobacco leaves. This, in turn, affects the synthesis of nitrogen-containing compounds like proteins and chlorophyll, potentially hindering tobacco leaf growth and development, resulting in decreased quality and negatively influencing crop yield (Prasad et al., 2008; Ahanger et al., 2016).

Under combined heat and drought stress, chlorophyll content and the activities of SOD and POD enzymes in tobacco leaves were significantly lower than under heat or drought stress alone, while relative conductivity and MDA content were significantly higher. It indicates that the synergistic stress of heat and drought causes more significant harm to tobacco compared to individual stressors (Wang et al., 2024). Severe drought at different stages of tobacco growth and development affects leaf quality, including changes in the chemical composition and aroma substances (Bahrami-Rad and Hajiboland, 2017). Studies showed that mild drought stress during the maturity stage is detrimental to forming and transforming phenols, higher fatty hydrocarbons, and flavonoids, leading to decreased aroma and taste quality of cured tobacco leaves (Begum et al., 2021). In response to the synergistic stress of heat and drought, tobacco’s ecological adaptation mechanisms are primarily reflected in adjusting the plant’s physiological structure to adapt to adverse environments, such as increasing leaf cuticle thickness and reducing leaf surface area to minimize water loss and altering biochemical pathways to enhance stress tolerance efficiency, such as increasing the activity of antioxidant enzymes, such as CAT and SOD to reduce oxidative stress caused by heat and drought (Carmo-Silva et al., 2012).

## Influence of genes expression and signal transduction

Heat and drought stress significantly disrupt protein homeostasis within plant cells, leading to a cascade of physiological and biochemical responses (Feller and Vaseva, 2014). Under heat stress conditions, the structural integrity and properties of tobacco plant cell walls are compromised. This increases membrane fluidity and permeability, facilitating the efflux of extracellular calcium ions (Ca^2+^), a crucial signaling molecule in various cellular processes (Niu and Xiang, 2018).The elevation of cytosolic Ca^2+^ levels activates a series of downstream signaling pathways. Concurrently, reactive oxygen species (ROS) and nitric oxide (NO) emerge as critical secondary messengers in response to heat stress. These molecules mediate stress responses by rapidly activating regulatory networks that influence gene expression and metabolic adjustments. The accumulation of ROS can trigger oxidative stress, damaging cellular components, while NO promotes stress tolerance and signaling pathways (Goraya et al., 2017).Heat stress results in the overproduction of cytosolic Ca^2+^, ROS, and NO, disrupting the endoplasmic reticulum’s protein-folding capacity (ER). This leads to the accumulation of misfolded or unfolded proteins, a condition known as ER stress. Such proteins can exert toxic effects on cellular functions and ultimately compromise cell viability. The accumulation of misfolded proteins triggers the unfolded protein response (UPR), a protective mechanism to restore protein homeostasis and mitigate cellular damage. In response to these imbalances, plants have evolved various adaptive mechanisms to cope with heat stress and minimize cellular damage. These include the upregulation of heat shock proteins (HSPs), which assist in protein folding and stabilization, and the activation of antioxidant systems to counteract ROS-induced damage. However, plants may alter metabolic pathways to enhance osmoregulation and improve stress resilience (Chaudhry and Sidhu, 2022). Plants strive to maintain cellular integrity and functionality through these complex responses in adverse environmental conditions.

In response to heat stress, tobacco plants activate multiple heat stress-responsive genes, typically regulated by heat shock transcription factors (HSFs) (Dos Santos et al., 2022). HSFs are key regulators in plants’ rapid response to heat stress. It binds to the heat shock proteins (HSPs) gene promoter regions, initiating their expression. HSPs function as molecular chaperones, assisting in proper protein folding, preventing protein aggregation and denaturation caused by heat, and thus protecting cellular protein function (Becker and Craig, 1994). In tobacco, genes like *HSP70* and *HSP90* have been extensively studied, and their upregulated expression under heat stress plays a significant role in maintaining cellular homeostasis and enhancing heat tolerance efficiency (Li et al., 2015).

However, genes associated with antioxidative responses, such as SOD and CAT, are also induced under heat-stress conditions. These genes protect cells from oxidative damage by scavenging ROS, enhancing tobacco’s stress tolerance (Devireddy et al., 2021). Studies have shown that the overexpression of specific heat shock protein genes, like*ZmHSP16.9*, *RcHSP17.8*, *BcHSP70, and LeHSP21*, can maintain photosynthetic responses by increasing seed germination rates, chlorophyll content, and antioxidant capacity while reducing MDA content and conductivity, thereby significantly enhancing plant heat tolerance apparatus (Sun et al., 2012). Furthermore, overexpression of transcription activator genes like *CAP2*, seed dormancy delay family gene NtDOG1L-T, and heat shock transcription factor gene *BcHsfA1* in tobacco seedlings significantly enhances promoter activity under heat stress conditions, with notable increases in the expression levels of HSPs and heat shock factor genes (Zhu et al., 2018). Research has also noticed that N-acetyl glutamate kinase (NAGK) enhances plant heat tolerance by activating antioxidant defense signals such as ascorbate peroxidase 2 (*APX2*) and superoxide dismutase C (SODC), as well as the heat shock network genes.

Drought stress triggers the activation of abscisic acid (ABA)-dependent and independent signaling pathways in tobacco plants, which regulate the expression of related genes (Soma et al., 2021). ABA is a key hormone in plant response to drought stress. Under drought conditions, the synthesis and accumulation of ABA significantly increase, activating the ABA-dependent signaling pathway (Muhammad Aslam et al., 2022). ABA binds to its receptors, such as the PYR/PYL/RCAR family proteins, inhibiting the activity of PP2C phosphatases and releasing the inhibition of SnRK2 protein kinases. Once activated, SnRK2 kinases can phosphorylate and activate a series of downstream target proteins, including transcription factors, ion channels, and metabolic enzymes, thereby initiating the expression of drought-resistant genes (Kobayashi et al., 2005; Zhu, 2016). ABA can activate ABA-responsive element (ABRE)-binding proteins, including transcription factors like ABF/AREB, which regulate the expression of drought-resistant genes. These genes often encode proteins related to drought resistance, such as LEA proteins, aquaporins, and osmotic regulatory proteins, helping plants maintain cellular homeostasis and water balance (Yoshida et al., 2010; Soma et al., 2021).

Reactive oxygen species (ROS) act as signaling molecules and damaging agents in plant stress responses (Mittler et al., 2022). The production of ROS typically increases under temperature and drought stress, triggering a series of stress responses (Czarnocka and Karpiński, 2018). ROS can initiate signal transduction by oxidatively modifying specific proteins, altering their function or activity. For example, ROS can activate the MAPK (mitogen-activated protein kinase) cascade, amplifying stress signals and promoting downstream responses (Jalmi and Sinha, 2015). Under the regulation of ROS signaling, plants activate the expression of antioxidant genes, such as those encoding SOD, CAT and APX. These enzymes mitigate oxidative stress by scavenging excess ROS, thereby protecting cellular structure and function (Azarabadi et al., 2017).

Studies have shown that Late Embryogenesis Abundant (LEA) protein genes in tobacco are significantly upregulated under drought stress. The proteins encoded by these genes stabilize cellular membranes and proteins, reducing cell damage caused by drought (Bao et al., 2017). Dehydration-responsive Element Binding Protein (DREB) transcription factors play an essential role in the drought stress response in tobacco. DREB transcription factors can recognize and bind to the drought-responsive element (DRE) sequence, activating the expression of drought resistance-related genes, such as aquaporin and osmotic regulation-related genes. The products of these genes help tobacco cells maintain water balance and enhance drought tolerance capacity (Joshi et al., 2016; Siefritz et al., 2002).

## Mitigation strategies and prospects for heat and drought stress

### Agricultural management and cultivation techniques

Effective management of water and fertilizers plays a crucial role in influencing tobacco leaf yield and structure (Li et al., 2009). Implementing appropriate irrigation practices can mitigate soil temperature under elevated thermal conditions, simultaneously enhancing the uptake of essential nutrients such as nitrogen, phosphorus, and potassium. This, in turn, increases the activity of key enzymes involved in carbon and nitrogen metabolism, thereby alleviating the detrimental effects of heat stress on tobacco plants (Ferrante and Mariani, 2018; Kurt and Kinay, 2021).Elements such as silicon and potassium act as regulatory elements that can enhance plant growth and increase resistance to stress. Research shows that the increasing potassium fertilizer application can stabilize the plasma membrane, reduce membrane permeability, and reduce leaf wilting efficiency, thereby effectively enhancing tobacco resistance (Kumari et al., 2022).

Moreover, applying appropriate amounts of calcium and magnesium fertilizers under heat-stress conditions can significantly improve tobacco stress conditions (Waraich et al., 2012). Calcium maintains the structural and functional integrity of cell membranes and acts as a secondary messenger in plant responses to the adaptation of environmental variables (Thor, 2019). Under heat stress, exogenous calcium and calcium chloride can effectively regulate photosynthesis in tobacco, improve stomatal conductance in plant leaves, enhance the thermal stability of the oxygen-evolving complex, and reduce ROS accumulation, thus alleviating heat stress (Tan et al., 2011).

### Genetic engineering strategies

Genetic engineering provides essential avenues for mitigating heat and drought stress in tobacco plants. Researchers can precisely regulate stress-responsive genes through gene editing technologies such as CRISPR/Cas9 (Doudna and Charpentier, 2014). For example, overexpressing antioxidant enzyme genes or osmotic regulatory substance synthesis genes can enhance the ability of tobacco to scavenge ROS, thereby improving cellular osmotic protection. However, regulating essential genes such as transcription factors like DREB and HSF can further activate downstream stress response genes, enhancing tobacco’s stress resistance capacity (Manna et al., 2021). However, genetic engineering approaches still face challenges regarding public acceptance, ecological safety and regulatory policies.

### Application of biotechnology

Biotechnology shows great promise in improving tobacco’s tolerance to heat and drought stress conditions. For example, treating tobacco with endophytes or microbial inoculants can enhance its stress resistance. Studies have found that certain endophytic fungi or bacteria can improve tobacco’s tolerance to environmental stress by regulating the plant’s hormonal balance, increasing antioxidant capacity, or enhancing osmotic regulation (Begum et al., 2022). The use of plant growth regulators, such as abscisic acid analogues (ABA analogues), to induce stress responses in plants is an effective strategy (Wang et al., 2024).

### Molecular breeding and germplasm innovation

Molecular breeding techniques, such as selecting and cultivating tobacco varieties with high resistance to heat and drought, represent a long-term strategy for coping with the era of climate change. Technologies like genome-wide association studies (GWAS) and genomic selection (GS) can be applied to identify quantitative trait loci (QTLs) associated with stress resistance and use them in marker-assisted breeding (Devate et al., 2022). Moreover, modern molecular tools, including transgenic technologies, gene editing and RNA interference, can accelerate the development of new stress-resistant germplasm. However, given the complexity and unpredictability of climate change, continuous exploration of recent stress-resistant traits and breeding strategies will be necessary in the future.

## Conclusion and future perspectives

High temperatures and drought represent significant environmental stressors that profoundly influence the growth and development of tobacco plants. These stressors adversely affect both the yield and quality of tobacco post-curing. A comprehensive understanding of the mechanisms by which high temperature and drought exert their effects is essential for implementing effective preventive measures in field demonstrations. Future research should prioritize the exploration of strategies to mitigate these impacts. Moreover, attention should also be directed toward other stressors. For instance, concurrent high temperature and soil flooding stress during the late stages of tobacco growth in Yunnan, China, necessitates further investigation. In the context of climate change, developing strategies to regulate crop growth and development under optimal temperature conditions with limited water irrigation is becoming increasingly pertinent and holds significant implications for global agricultural security and sustainability. Despite the critical nature of these challenges, there is a scarcity of studies examining the development of root ultrastructure, rhizosphere microorganisms, and root exudates in response to the combined stress of optimal temperature and water scarcity. Therefore, intensified research efforts are essential to provide deeper insights into plant responses to high temperatures and drought by linking aboveground and belowground components. Understanding these interactions will be crucial as climate conditions are expected to become hotter and drier. Overall, this study systematically explores the development, trends, and prospects of plant responses to water stress and optimal temperature research, thereby enhancing our understanding of how plants adapt to the challenges posed by climate change.
